# Tumor-Infiltrating Lymphocytes (TILs) and Risk of a Second Breast Event After a Ductal Carcinoma *in situ*

**DOI:** 10.3389/fonc.2020.01486

**Published:** 2020-08-19

**Authors:** Alberto Farolfi, Elisabetta Petracci, Luigi Serra, Alessandra Ravaioli, Sara Bravaccini, Sara Ravaioli, Maria Maddalena Tumedei, Paola Ulivi, Matteo Canale, Maurizio Puccetti, Fabio Falcini, Secondo Folli, Annalisa Curcio, Andrea Rocca

**Affiliations:** ^1^Department of Medical Oncology, Istituto Scientifico Romagnolo per lo Studio e la Cura dei Tumori (IRST) IRCCS, Meldola, Italy; ^2^Unit of Biostatistics and Clinical Trials, Istituto Scientifico Romagnolo per lo Studio e la Cura dei Tumori (IRST) IRCCS, Meldola, Italy; ^3^Pathology Unit, Morgagni-Pierantoni Hospital, Forlì, Italy; ^4^Romagna Cancer Registry, Istituto Scientifico Romagnolo per lo Studio e la Cura dei Tumori (IRST) IRCCS, Meldola, Italy; ^5^Biosciences Laboratory, Istituto Scientifico Romagnolo per lo Studio e la Cura dei Tumori (IRST) IRCCS, Meldola, Italy; ^6^Pathology Unit, Azienda Unità Sanitaria Locale (AUSL), Imola, Italy; ^7^Fondazione IRCCS Istituto Nazionale dei Tumori, Milan, Italy; ^8^Senology Unit, Morgagni-Pierantoni Hospital, Forlì, Italy

**Keywords:** ductal carcinoma *in situ*, tumor infiltrating lymphocytes, second breast event, tumor recurrence, breast conserving surgery, radiotherapy

## Abstract

Women with a diagnosis of ductal carcinoma *in situ* (DCIS) have a high risk of developing a second breast event (SBE). The immune system might play a role in trying to prevent a SBE. Patients diagnosed with DCIS were identified in the population-based cancer registry of Area Vasta Romagna from 1997 to 2010. Median follow-up is 8.5 years. Tumor-infiltrating lymphocytes (TILs) were evaluated both in index DCIS and in SBE. The main endpoint was to assess the association between TILs' levels in index DCIS and risk of a SBE. Out of 496 DCIS patients, 100 SBEs (20.2%) were identified: 55 ipsilateral (11.1%) and 43 contralateral (8.7%). The distribution of TILs was heterogeneous, but significantly associated with grade, necrosis, screen detection and type of surgery. Patients stratified according to TILs percentage (≤5% and >5%) did not show a statistically significant difference in the 5-year cumulative incidence of SBEs: 14.9% (95% CI 11.3–19.1) and 11.0% (95% CI, 6.9–16.2), respectively (*p* = 0.147). In the subgroup of patients who did not receive radiotherapy, TILs >5% were associated with a reduced risk of SBE (HR 0.34, 95% CI 0.14–0.82, *p* = 0.016). Although we did not find any significant association between TILs and SBE, further studies evaluating their role according to radiotherapy are warranted.

## Background

Ductal carcinoma *in situ* (DCIS) is a malignant, clonal proliferation of cells growing within the basement membrane-bound structures of the breast and with no evidence of invasion into surrounding stroma (1). Although concern regarding the overtreatment of DCIS was recently raised (2), the association between screen-detected DCIS and subsequent invasive breast cancers suggests that detection and treatment of DCIS is worthwhile in prevention of future invasive disease and thus reduce breast cancer mortality (3, 4).

Patients with DCIS are at higher risk of a second breast cancer event (SBE), being it an *in situ* proliferation or an infiltrating carcinoma (1). Many factors impact on recurrence risk, (5) including patient age, tumor size, nuclear grade and margin width (6, 7). The relative importance of these factors remains controversial, and more recently complex nomograms (8), gene expression signatures (9), and proliferation markers (10) were evaluated to help in defining the risk of relapse, but are not used routinely yet.

A DCIS diagnosis increases the risk of a contralateral breast event (CBE), with almost twice the risk of the general population for a contralateral infiltrating carcinoma, independently of surgery, radiotherapy, age at diagnosis, histological subtype (comedocarcinoma vs. non-comedocarcinoma) and anatomical position of index DCIS (11). Hormonal therapy is the only factor having demonstrated to reduce the risk of CBE (12–14).

The increasing incidence of DCIS, likely to be sustained by the enhanced visualization that digital mammography provides, translates however in uncommonly recurrent DCIS or invasive cancers (4). In this context, validated prognostic factors for ipsilateral breast event (IBE) are few and even less if we consider CBE. Recently, tumor-infiltrating lymphocytes (TILs) proved to be associated with a better prognosis in HER2-positive and triple negative infiltrating breast carcinoma (15–18), and have been proposed as a reliable surrogate of the adaptive immune response. However, the issue of potential overtreatment of DCIS and the need of improvement in selection of adjuvant therapies remain unmet medical needs. In this context, we aimed to investigate the distribution and clinical implications of TILs in the DCIS setting regarding a SBE (both IBE and CBE).

## Patients and Methods

### Study Population

The study cohort consisted of all consecutive women diagnosed with incident DCIS between January 1st 1997 and December 31st 2010, identified through the “Area Vasta Romagna Cancer Registry” (R.T.Ro.). The reason for starting our observation time in 1997 is due to the fact that in 1997 started the mammography screening programme and so before this year data may be more fragmentary. R.T.Ro. was established in 1986 and since then it registers regularly all the cases of cancer diagnosed in the Romagna region, covering a population of 1.159.218 inhabitants. In order to retrieve information on SBEs and death, registry data were integrated with an extended medical chart review until August 30th 2015.

*In situ* breast carcinomas were identified using D051, D057, and D059 codes according to the 10th edition of the International Classification of Diseases (ICD-10). In order to identify only DCIS, histology of breast cancer based on the 3rd edition of the International Classification of Diseases for Oncology (ICD-O-3) was also used: ductal carcinoma *in situ* (8500/2, 8501/2, 8503/2). In accordance with ICD-O-3, diagnosis of 8520/2, corresponding to lobular neoplasms *in situ*, were excluded. Other exclusion criteria for the present study were: previous diagnosis of a breast cancer (DCIS or invasive, antecedent to the index DCIS), presence of microinfiltrating or infiltrating ductal carcinoma, synchronous bilateral breast cancer, and a diagnosis of a SBE within 6 months from the surgical removal of the index DCIS.

The present study was approved by the Ethics Committee of Romagna C.E.ROM. (Comitato Etico IRST IRCCS-AVR CEIIAV), with approval number 1166 of 17 July 2014; was also conducted in accordance with the Declaration of Helsinki and all patients signed the informed consent.

### TIL-Assessment

**Figure 1:**
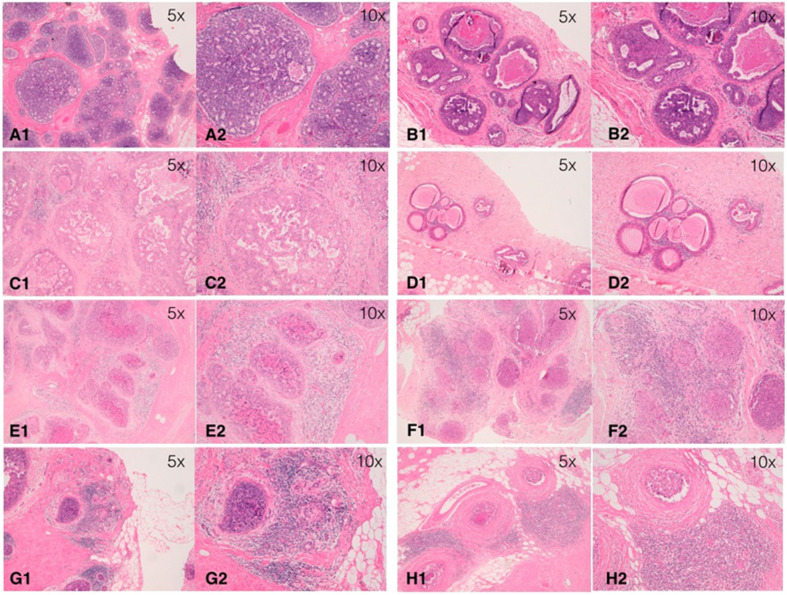
Examples of distribution of tumor-infiltrating lymphocytes (TILs) on hematoxylin-eosin section of ductal carcinoma *in situ* (DCIS) at different magnifications (5x and 10x). **(A)** TIL = 0%; **(B)** TIL = 1%; **(C)** TIL = 5%; **(D)** TIL = 10%; **(E)** TIL = 20%; **(F)** TIL = 40%; **(G)** TIL = 50%; **(H)** TIL = 70%.

In this study, stromal TILs were scored using a method based on the International Immuno-Oncology Biomarker Working Group guidelines for invasive breast carcinoma (19), with some modifications specific to the DCIS setting (20). In particular, stromal area was defined as the specialized stroma surrounding the ducts involved by *in-situ* carcinoma. Any type of circumferential infiltrate (only mononuclear cells, but not polymorphonuclear leukocytes) was taken into account, including minimal, partial, subtotal, and total circumferential TILs. TILs in tumor zones with crush artifacts, necrosis, regressive hyalinization as well as in the previous core biopsy site were excluded. TILs were assessed as a continuous parameter as the percentage of stromal area covered by mononuclear cells (Figure 1). Levels of TILs on index DCIS were examined for their associations with SBE as the primary endpoint. Subsequently, TILs distribution was assessed in SBEs and compared to the levels on the primary lesion in order to evaluate its variation.

### Statistical Methods

Data were summarized by using mean ± standard deviation or median and minimum and maximum values or interquartile range, as appropriate, for continuous variables and by means of frequencies and percentages for categorical ones. The percentage of TILs was considered either as a continuous variable or a binary one (≤5% vs. >5%). As no validated cut-off value for DCIS patients was present at the time of analyses, we decided to consider the one based on the median value of TILs distribution. Due to the quite right-skewed distribution, we decided to not consider other quantiles. The Chi-square test or the Fisher Exact test, when appropriate, and the Wilcoxon-Mann Whitney test were used to compare patients' characteristics between the two groups of patients defined by TILs. The primary end-point of this study was the time since surgery to any type of SBE. The median follow-up time was computed as the median time in study for patients not experiencing any event. Other secondary end-points were the time to a second ipsilateral breast event and the time to a contralateral breast event. For the first secondary end-point, other first events such as contralateral breast cancers, other primary tumors and deaths from causes other than breast cancer were considered as competing events. For the other secondary end-point, ipsilateral breast cancers, other primary tumors and deaths from causes other than breast cancer were considered as competing events. Five-year cumulative incidences were computed for SBE, IBE, and CBE and the Gray's test was used to compare them between groups of patients. The Cox proportional hazards model was used to evaluate the effect of TILs and other covariates on the risk of SBE. The inspection of the functional form for TILs was performed through martingale residuals and by means of restricted cubic splines with three knots. To test for departure from a linearity, the Likelihood Ratio Test was used comparing a model with only a linear term for TILs and one including splines. For all the analysis a two-sided *p*-values < 0.05 was considered statistically significant. All analyses were carried out with STATA 14.0 (College Station, Texas, USA) and R version 3.4.0 statistical software (http://cran.r-project.org/).

## Results

### Patient Characteristics

**Figure 2:**
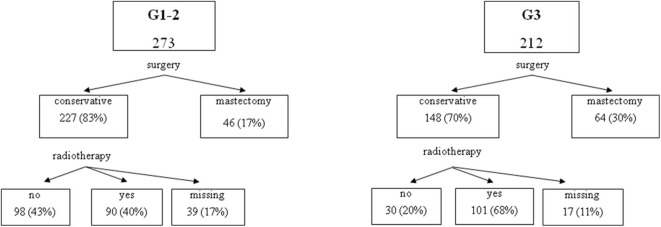
Distribution of patients by grade and treatment.

A total of 496 cases of DCIS were included in this study. Table 1 shows baseline characteristics for the entire cohort. Median patients age at diagnosis was 56.4 years (range 27.3–89.3 years). The majority of patients had a conservative surgery (either nodulectomy or quadrantectomy: 77.0%), whereas 114 patients (23.0%) were treated with mastectomy. Radiotherapy was performed in 59.7% of the patients treated with conservative surgery. None of the patients received adjuvant endocrine therapy. Figure 2 presents the distribution of patients' treatments according to the DCIS grade.

**Table 1:** Main baseline characteristics of the 496 DCIS patients included in the study.

**Patients characteristics**		**TILs**					
	**All patients**		**≤5%**		**>5%**		***p*****-value**
	**No. 496**		**No. 323**		**No. 173**		
**Age at diagnosis, years**	No.	%	No.	%	No.	%	
Median [min–max]	56.4 [27.3–89.3]		54.8 [30.3–89.3]		57.8 [27.3–84.3]		0.309
**Surgery**							
Conservative surgery	382	77.0	258	79.9	124	71.7	0.039
Mastectomy	114	23.0	65	20.1	49	28.3	
**Grade**							
G1–2	273	56.3	215	68.0	58	34.3	<0.001
G3	212	43.7	101	32.0	111	65.7	
Missing	11		7		4		
**Necrosis**							
Absent	205	44	158	52.0	47	29.0	<0.001
Present	261	56	146	48.0	115	71.0	
Missing	30		19		11		
**Margins**							
Negative	393	90.8	263	90.4	130	91.6	0.693
Close/positive	40	9.2	28	9.6	12	8.4	
Missing	63		32		31		
**No. excisions**							
1	445	91.4	289	91.8	156	90.7	0.694
≥2	42	8.6	26	8.3	16	9.3	
Missing	9		8		1		
**Screen detected**							
No	294	59.5	203	63.0	91	52.9	0.029
Yes	200	40.5	119	37.0	81	47.1	
Missing	2		1		1		
**Radiotherapy**^†^							
No	129	40.3	95	43.4	34	33.7	0.100
Yes	191	59.7	124	56.6	67	66.3	
Missing	62		39		23		

† *Numbers refer to women having a conservative surgery*.

In primary DCIS, median TILs level was 5% (range 0–90%, the first and fourth quartiles were 0 and 10%) with a heterogeneous distribution across the 496 DCIS analyzed: 187 (37.7%) had <1%, 276 (55.6%) 1–49% and 33 (6.7%) ≥50% stromal lymphocytes infiltration. Median TILs levels in primary tumors were higher in patients treated with mastectomy, in those with a high grade DCIS or with necrosis and in those receiving radiotherapy (Table 2). Similarly, factors significantly associated with TILs, when dichotomized between ≤5 and >5%, were grade, necrosis, type of surgery, and detection mode (within or outside the mammography screening program) as shown in Table 1.

**Table 2:** Median TILs distribution per main characteristic.

**Patients characteristics**	**TILs**	***p-*value**
	**Median (%) [min–max]**	
**Age at diagnosis, years**		
<56.4	1 [0–90]	0.518
≥56.4	5 [0–80]	
**Surgery**		
Conservative surgery	1 [0–90]	0.022
Mastectomy	5 [0–80]	
**Grade**		
G1–2	0 [0–90]	<0.001
G3	10 [0–80]	
**Necrosis**		
Absent	0 [0–80]	<0.001
Present	5 [0–90]	
**Margins**		
Negative	1 [0–80]	0.635
Close/positive	1 [0–40]	
**No. excisions**		
1	2 [0–90]	0.993
≥2	5 [0–60]	
**Screen detected**		
No	1 [0–80]	0.068
Yes	5 [0–90]	
**Radiotherapy**^†^		
No	0 [0–90]	0.027
Yes	1 [0–80]	

† *Numbers refer to women having a conservative surgery*.

### Association Between TILs and Second Breast Cancer Events (SBEs)

After a median follow-up of 8.5 years, a total of 100 (20.2%) SBEs occurred, 53 (10.7%) of which were ipsilateral (IBE): 16 *in situ* (3.2%) and 37 invasive (7.5%), and 45 (9.1%) contralateral (CBE): 14 *in situ* (2.8%) and 31 invasive (6.3%). For two cases it was not possible to identify if the SBE was ipsilateral or contralateral because laterality information was missing. Overall, 35 (7.1%) other events occurred (30 s non-breast primaries, 5 deaths for non-breast related causes).

**Figure 3:**
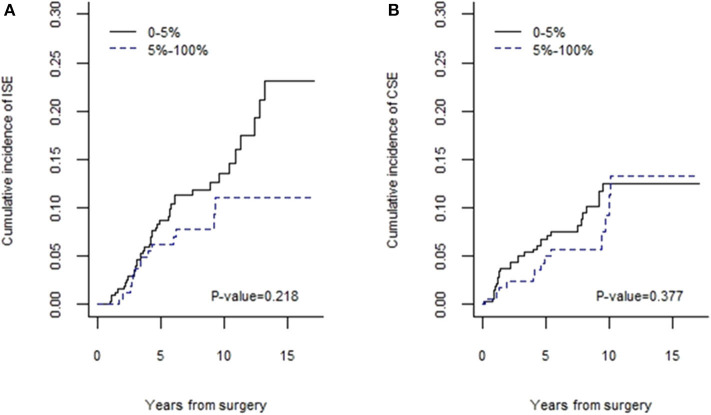
Cumulative incidences of IBEs **(A)** and CBEs **(B)**.

Patients stratified according to TILs percentage (≤5 and >5%) did not show a statistically significant difference in the 5-year cumulative incidence of *in situ* and invasive SBEs: 14.9% (95% CI 11.3–19.1) for TILs ≤5% and 11.0% (95% CI 6.9–16.2) for TILs >5% (*p* = 0.147). DCIS with TILs ≤5% showed a 5-year cumulative incidence of IBEs of 8.4% (95% CI 5.6–11.9), vs. 6.1% (95% CI 3.1–10.6) for those with TILs >5% (*p* = 0.218, Figure 3). Again, 5-year cumulative incidence of CBEs was 7.5% (95% CI 4.9–10.8) and 5.0% (95% CI 2.3–9.2) for a lymphocyte infiltration ≤5 and >5%, respectively (*p* = 0.377, Figure 3).

As shown in Table 3, factors significantly associated with SBEs at univariate analysis were type of surgery (associated also with IBEs) and grade. Although a high grade seems to be associated with a reduced risk of SBEs in this analysis, likely due to the preferential treatment with radiotherapy in G3 cases, the effect of grade is reversed when considering the subgroup of patients who underwent conservative surgery with negative resection margins and who did not receive radiotherapy (HR = 1.04, 95% CI 0.4–2.6, *p* = 0.928). The effect of TILs as a continuous variable was negligible on the risk of SBEs, IBEs and CBEs. A 10% increase in TILs was associated with a relative risk reduction of 2 and 7% for SBEs and IBEs, respectively, and with a 4% relative risk increase for CBEs. No statistically significant departure from linearity was observed for the relationship of TILs and SBE (*p* = 0.778), IBE (*p* = 0.921), and CBE (*p* = 0.716). Even when cut-off values were considered, no statistically significant association was observed (results not shown). This was confirmed even after adjustment for other clinical covariates.

**Table 3:** Univariate analysis of the risk of second breast events (SBEs), ipsilateral (IBEs), and controlateral (CBEs) breast events (*in situ* or invasive).

	**SBE**			**IBE**			**CBE**		
	**HR**	**(95% CI)**	***p-*value**	**HR**	**(95% CI)**	***p-*value**	**HR**	**(95% CI)**	***p-*value**
**Age at diagnosis, yrs**^†^	1.14	(0.95–1.36)	0.147	1.20	(0.95–1.53)	0.133	1.06	(0.81–1.39)	0.669
**Surgery**									
Conservative	1			1			1		
Mastectomy	0.37	(0.19–0.71)	0.003	0.19	(0.06–0.62)	0.006	0.64	(0.29–1.44)	0.282
**Radiotherapy**^§^									
No	1			1			1		
Yes	0.96	(0.61–1.49)	0.852	1.33	(0.74–2.39)	0.349	0.62	(0.30–1.28)	0.196
**Grade**									
G1–2	1			1			1		
G3	0.56	(0.37–0.86)	0.008	0.62	(0.35–1.09)	0.095	0.48	(0.25–0.94)	0.032
**Necrosis**									
Absent	1			1			1		
Present	1.11	(0.73–1.67)	0.610	1.13	(0.66–1.94)	0.663	1.15	(0.62–2.16)	0.656
**Margins**									
Negative	1			1			1		
Close or positive	1.73	(0.92–3.26)	0.092	1.61	(0.69–3.80)	0.273	1.91	(0.74–4.94)	0.180
**No. excisions**									
1	1			1			1		
≥2	1.37	(0.74–2.58)	0.317	1.62	(0.74–3.61)	0.228	1.11	(0.40–3.11)	0.841
**Screen detected**									
No	1			1			1		
Yes	0.70	(0.46–1.06)	0.093	0.70	(0.40–1.23)	0.220	0.60	(0.31–1.16)	0.128
**TILs**^‡^	0.98	(0.87–1.11)	0.797	0.93	(0.78–1.12)	0.443	1.04	(0.88–1.23)	0.635
**TILs**									
≤5%	1			1			1		
>5%	0.60	(0.24–1.38)	0.211	0.56	(0.16–1.95)	0.363	0.63	(0.18–2.19)	0.463

§ *Estimates refer to women having a conservative surgery*.

† *Expressed as 5-year increase*.

‡ *Expressed as 10% increase*.

In the subgroup of patients who did not receive radiotherapy (225 patients), TILs, when considered as a continuous variable, were not associated with any type of SBE. Hazard ratios for a 10% TILs increase were 0.93 (95% CI 0.75–1.15; *p* = 0.504), 0.95 (95% CI 0.70–1.29; *p* = 0.742), and 0.93 (95% CI 0.69–1.25; *p* = 0.618) for SBE, IBE, and CBE, respectively. In the same subgroup, when considered as categorical variable, TILs >5% were associated with a reduced risk of SBE (HR 0.33, 95% CI 0.14–0.79, *p* = 0.013). This effect remained significant also after adjustment for grade and margins at a multivariate analysis (adjusted HR 0.31, 95% CI 0.11–0.93, *p* = 0.037).

The effect was lost when considering the risk for IBEs (HR 0.36, 95% CI 0.10–1.23, *p* = 0.104) and for CBEs (HR 0.33, 95% CI 0.10–1.11, *p* = 0.075) separately, presumably due to the smaller sample size. In the complementary subgroup of women treated with conservative surgery and radiotherapy, no statistically significant associations were observed between TILs values, both when considered as a continuous and categorical variable, and the risk of SBEs, IBEs, or CBEs.

### TIL Count in SBEs

**Figure 4:**
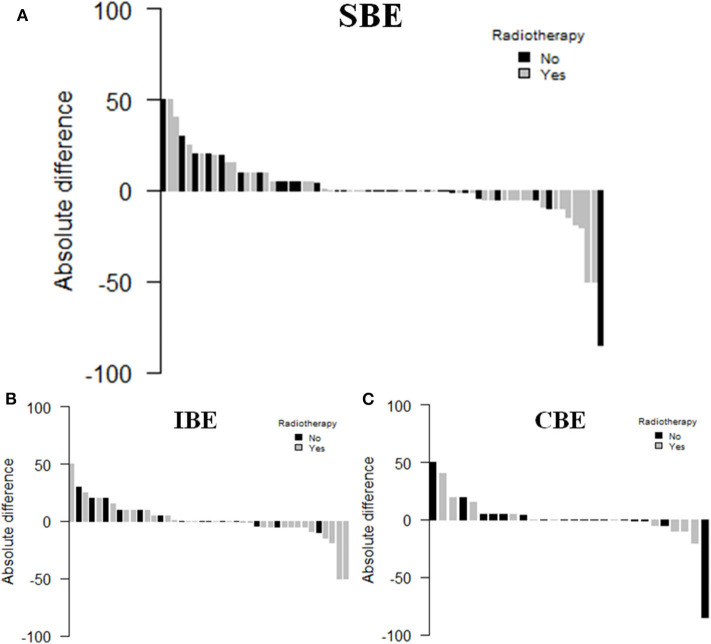
Waterfall plot of TILs variation between primary DCIS and SBE **(A)**, IBE **(B)**, or CBE **(C)**.

Out of 100 SBEs, 77 patients, 45 with a IBE (58.4%) and 32 with a CBE (41.6%), had paraffin embedded breast cancer tissue available and were thus evaluated for TILs. Table 4 shows the main clinical characteristics of the patients with matched SBEs. The median TILs levels in primary DCIS and matched SBE were 1% (range 0–90%) and 5% (range 0–80%), respectively (*p* = 0.971). In the subgroup of patients treated with radiotherapy, median TILs levels in primary DCIS and matched SBE were 5% (range 0–50%) and 0.5% (range 0–80%), respectively (*p* = 0.720). DCIS not treated with radiotherapy showed an increase in median TILs levels: 0 (range 0–90%) and 5% (range 0–70%), respectively (*p* = 0.359). Figure 4 shows the waterfall plots of absolute differences (TILs of the SBE–TILs on the primary tumor) in TILs according to radiotherapy treatment.

**Table 4:** Main baseline characteristics of the 77 DCIS patients with matched SBEs.

**Patients characteristics**	**No**.	**(%)**
**Age at diagnosis**		
Median [min–max]	57.6 [35.5–85.2]	
**Surgery**		
Conservative surgery	71	(92.2)
Mastectomy	6	(7.8)
**Radiotherapy**		
No	31	(44.9)
Yes	38	(55.1)
Missing	8	
**Grade**		
G1–2	51	(67.1)
G3	25	(32.9)
Missing	1	
**Necrosis**		
Absent	31	(43.1)
Present	41	(56.9)
Missing	5	
**Margins**		
Negative	60	(85.7)
Close/positive	10	(14.3)
Missing	7	
**No. excisions**		
1	69	(89.6)
≥2	8	(10.4)
**Screen detected**		
No	53	(69.7)
Yes	23	(30.3)
Missing	1	
**TILs**		
Median [min–max]	0 [0–1]	
**TIL**		
≤5%	57	(74.0)
>5%	20	(26.0)

## Discussion

TILs seem to represent an easy and reliable prognosticator in HER2 positive and triple negative infiltrating breast carcinoma (15–18). Data on their role in DCIS are more scanty, with conflicting results in terms of prognostic values (21, 22). In our study, evaluating a large series of consecutive patients extracted from a cancer register, we used criteria for scoring stromal lymphocytes in the DCIS setting according to a modified method based on the International Immuno-Oncology Biomarker Working Group guidelines for invasive breast carcinoma (19), concordant with the more recent recommendations (20). The validity of our evaluation was confirmed considering the distribution of TILs per category, virtually identical to the results obtained by Pruneri and co-workers, and the same association of TILs with grade and necrosis (21). Similarly, we did not find any significant association among TIL levels and the risk of IBEs, although the incidence was numerically higher in cases with low TILs, and we cannot exclude that a larger sample size could have shown significant differences.

Moreover, in our study, we analyzed for the first time the association with contralateral events, but no significant association was demonstrated with CBEs nor with all SBEs. Finally, we first reported an evaluation on the change in the distribution of TILs between index DCIS and SBEs, showing a decrease in the TILs number in the secondary events of patients previously treated with radiotherapy for their index DCIS. Although not statistically significant, this deserves further research.

The relationship between TILs and aggressive DCIS features is somehow in contrast with the observation, in our study, of a lack of association between TILs and SBEs. Accordingly, other papers reported the absence of an association with ipsilateral and controlateral *in situ* or invasive tumor recurrence (21, 23, 24). TILs could have a protective effect at least in some cases of high-risk DCIS, counteracting the negative impact of factors such as high grade and necrosis, but this remains to be definitely proven.

Interestingly, we observed that in the subgroup of patients not treated with radiotherapy, TILs >5% were associated with a reduced risk of SBEs. More exploratory is the observation of a reduction in TILs in the matched SBEs induced by radiotherapy, although not statistical significant.

No data on the effect of radiotherapy on TILs in breast cancer exist, but in cervical cancer dynamic changes of TILs after local irradiation have been reported. Interestingly, the sensitivity of the various lymphocyte populations to ionizing radiation *in vivo* was variable: the tumor infiltrating FOXP3+ Treg cells were more resistant compared with CD8+ T cells, and may dominate the tumor milieu after radiotherapy (25). On the contrary, neoadjuvant chemotherapy for cervical cancer resulted in a decreased FOXP3+ density, resulting in an elevated intratumoral CD8/FOXP3 ratio that might confer a favorable clinical outcome (26). Similar results were shown also in rectal cancer with neoadjuvant chemoradiotherapy (27–29). Accordingly, high TILs have been demonstrated to be associated with a survival benefit only in HER2-positive or triple negative breast cancer patients treated with chemotherapy, alone or in combination with trastuzumab, but not in ER-positive, HER2-negative breast cancer (30). More studies on the effect of radiotherapy on breast cancer and on DCIS are needed to better understand the strict interplay between immune-system and ionizing radiations and if this would be translated in a clinical application.

The cross-talk between leukocytes, tumoral microenvironment and tumor cells is more complex. Hence, a limitation of our study is the restriction of our analysis to TILs scoring on H&E only, without assessing the relative density of specific subpopulations of cells and the functional status of the immune infiltrate. Since it has been reported that leukocytes subpopulations are associated with the prognosis of breast cancer patients independently of the absolute TILs value (24), it is possible that important associations may have been missed. For instance, higher numbers of tumor infiltrating B-lymphocytes (TIL-B) were associated with shorter recurrence-free interval in patients with DCIS (24).

None of the patients in our cohort received adjuvant endocrine therapy after surgical removal of their index DCIS, according to the local guidelines followed in the period of time covered by the study, and related to the lack of a clear demonstration of an overall survival benefit with systemic therapy. The NCCN guidelines still suggest that, since a survival advantage has not been demonstrated, the use of adjuvant systemic therapy for DCIS should be based on individual consideration of risk and benefit. Nonetheless, the protective role of an elevated level of TILs, if confirmed in larger cohorts, could acquire even more relevance in patients treated with conservative surgery plus adjuvant endocrine therapy, where it could contribute to select patients who could be spared radiotherapy.

Other important limitations of the current study include the retrospective design and the potential presence of confounding factors or indication bias, difficult to control with the available information. The lack of important variables such as tumor size, hormone receptor and HER2 status, proliferation markers and genetic features could have reduced the possibility to identify subgroups with differences in the risks of SBEs. Limitations in sample size may have conditioned the power to detect significant differences among subgroups and interactions among factors. However, even if other studies demonstrated an association between the distribution of TILs and DCIS subtypes, TILs in HER2 positive and triple negative DCIS were not associated with ipsilateral recurrence (21).

## Conclusions

This study confirmed the previous observations of the associations of TILs with grade and necrosis. We observed also an interesting role as prognostic factor in patients not treated with radiotherapy. Expansion of this work on a larger series of patients is warranted, to confirm these preliminary data.

## Data Availability Statement

The datasets generated for this study are available on request to the corresponding author.

## Ethics Statement

The present study was approved by the local Ethics Committee Area Vasta Romagna and IRST with approval number 1166 of 17 July 2014. The patients/participants provided their written informed consent to participate in this study.

## Informed Consent

Informed consent was obtained from all individual participants included in the study.

## Author Contributions

AF contributed the study concept. AF and EP contributed the study design. LS, SB, SR, MT, PU, MC, and MP performed all laboratory and pathological analyses including immunohistochemistry. ARa, FF, SF, and AC provided patients data. EP performed the statistical analysis. AF, EP, and ARo discussed data interpretation. AF wrote the article. EP and ARo revised the article. All authors read and approved the final manuscript. All authors contributed to the article and approved the submitted version.

## Conflict of Interest

The authors declare that the research was conducted in the absence of any commercial or financial relationships that could be construed as a potential conflict of interest.

## References

[1] Pinder SE. Ductal carcinoma *in situ* (DCIS): pathological features, differential diagnosis, prognostic factors and specimen evaluation. Mod Pathol. (2010) 23:S8–13. 10.1038/modpathol.2010.40 20436505

[2] Independent UK Panel on Breast Cancer Screening The benefits and harms of breast cancer screening: an independent review. Lancet. (2012) 380:1778–86. (2012) 10.1016/S0140-6736(12)61611-0 23117178

[3] Duffy SW Dibden A Michalopoulos D Offman J Parmar D Jenkins J. Screen detection of ductal carcinoma *in situ* and subsequent incidence of invasive interval breast cancers: a retrospective population-based study. Lancet Oncol. (2016) 17:109–114. 10.1016/S1470-2045(15)00446-5 26655422 PMC4691349

[4] Thompson AM Clements K Cheung S Pinder SE Lawrence G Sawyer E. Management and 5-year outcomes in 9938 women with screen-detected ductal carcinoma *in situ*: the UK sloane project. Eur J Cancer. (2018) 101:210–9. 10.1016/j.ejca.2018.06.027 30092498

[5] Toss A Palazzo J Berger A Guilesa F Sendeckie JA Simone N. Clinical-pathological features and treatment modalities associated with recurrence in DCIS and micro-invasive carcinoma: who to treat more and who to treat less. Breast. (2016) 29:223–30. 10.1016/j.breast.2016.07.023 27506636

[6] Benson JR Wishart GC Predictors of recurrence for ductal carcinoma in situ after breast-conserving surgery. Lancet Oncol. (2013) 14:348–57 10.1016/S1470-2045(13)70135-9 23896274

[7] Maxwell AJ Clements K Hilton B Dodwell DJ Evans A Kearins O. Risk factors for the development of invasive cancer in unresected ductal carcinoma *in situ*. Eur J Surg Oncol. (2018) 44:429–35. 10.1016/j.ejso.2017.12.007 29398324

[8] Rudloff U Jacks LM Goldberg JI Wynveen CA Brogi E Patil S. Nomogram for predicting the risk of local recurrence after breast-conserving surgery for ductal carcinoma *in situ*. J Clin Oncol. (2010) 28:3762–9. 10.1200/JCO.2009.26.8847 20625132

[9] Solin LJ Gray R Baehner FL Butler SM Hughes LL Yoshizawa C. A multigene expression assay to predict local recurrence risk for ductal carcinoma *in situ* of the breast. J Natl Cancer Inst. (2013) 105:701–10. 10.1093/jnci/djt067 23641039 PMC3653823

[10] Lazzeroni M Guerrieri-Gonzaga A Botteri E Leonardi MC Rotmensz N Serrano D. Tailoring treatment for ductal intraepithelial neoplasia of the breast according to Ki-67 and molecular phenotype. Br J Cancer. (2013) 108:1593–601. 10.1038/bjc.2013.147 23579208 PMC3668474

[11] Habel LA Moe RE Daling JR Holte S Rossing MA Weiss NS. Risk of contralateral breast cancer among women with carcinoma *in situ* of the breast. Ann Surg. (1997) 225:69–75. 10.1097/00000658-199701000-00008 8998122 PMC1190608

[12] Wapnir I Dignam JJ Fisher B Mamounas EP Anderson SJ Julian TB. Long term outcomes of invasive ipsilateral breast tumour recurrences after lumpectomy in NSABP B-17 and B-24 randomised clinical trials for DCIS. J Natl Cancer Inst. (2011) 103:478–88. 10.1093/jnci/djr027 21398619 PMC3107729

[13] Cuzick J Sestak I Pinder SE Ellis IO Forsyth S Bundred NJ. Effect of tamoxifen and radiotherapy in women with locally excised ductal carcinoma *in situ*: long-term results from the UK/ANZ DCIS trial. Lancet Oncol. (2011) 12:21–9. 10.1016/S1470-2045(10)70266-7 21145284 PMC3018565

[14] Petrelli F Barni S. Tamoxifen added to radiotherapy and surgery for the treatment of ductal carcinoma *in situ* of the breast: a meta-analysis of 2 randomized trials. Radiother Oncol. (2011) 100:195–9. 10.1016/j.radonc.2011.02.005 21411161

[15] Loi S Sirtaine N Piette F Salgado R Viale G Van Eenooet F. Prognostic and predictive value of tumor-infiltrating lymphocytes in a phase III randomized adjuvant breast cancer trial in node-positive breast cancer comparing the addition of docetaxel to doxorubicin with doxorubicin-based chemotherapy: BIG 02-98. J Clin Oncol. (2013) 31:860–7. 10.1200/JCO.2011.41.0902 23341518

[16] Adams S Gray RJ Demaria S Goldstein L Perez EA Shulmanet LN. Prognostic value of tumor-infiltrating lymphocytes in triple-negative breast cancers from two phase III randomized adjuvant breast cancer trials: ECOG 2197 and ECOG 1199. J Clin Oncol. (2014) 32:2959–66. 10.1200/JCO.2013.55.0491 25071121 PMC4162494

[17] Denkert C von Minckwitz G Brase JC Sinn BV Gade S Kronenwettet R Tumor-infiltrating lymphocytes and response to neoadjuvant chemotherapy with or without carboplatin in human epidermal growth factor receptor 2-positive and triple-negative primary breast cancers. J Clin Oncol. (2015) 33:983–91. 10.1200/JCO.2014.58.1967 25534375

[18] Loi S Drubay D Adams S Pruneri G Francis PA Lacroix-Triki M. Tumor-infiltrating lymphocytes and prognosis: a pooled individual patient analysis of early-stage triple-negative breast cancers. J Clin Oncol. (2019) 37:559–69. 10.1200/JCO.18.01010 30650045 PMC7010425

[19] Salgado R Denkert C Demaria S Sirtaine N Klauschen F Pruneri G. The evaluation of tumor-infiltrating lymphocytes (TILs) in breast cancer: recommendations by an international TILs working group 2014. Ann Oncol. (2015) 26:259–71. 10.1093/annonc/mdu450 25214542 PMC6267863

[20] Hendry S Salgado R Gevaert T Russell PA John T Thapa B. Assessing tumor-infiltrating lymphocytes in solid tumors: a practical review for pathologists and proposal for a standardized method from the international immunooncology biomarkers working group: part 1: assessing the host immune response, TILs in invasive breast carcinoma and ductal carcinoma *in situ*, metastatic tumor deposits and areas for further research. Adv Anat Pathol. (2017) 24:235–51. 10.1097/PAP.0000000000000162 28777142 PMC5564448

[21] Pruneri G Lazzeroni M Bagnardi V Tiburzio GB Rotmensz N DeCensi A. The prevalence and clinical relevance of tumor-infiltrating lymphocytes (TILs) in ductal carcinoma *in situ* of the breast. Ann Oncol. (2017) 28:321–8. 10.1093/annonc/mdw623 28426105

[22] Toss MS Miligy I Al-Kawaz A Alsleem M Khout H Rida PC. Prognostic significance of tumor-infiltrating lymphocytes in ductal carcinoma *in situ* of the breast. Mod Pathol. (2018) 31:1226–36. 10.1038/s41379-018-0040-8 29559742

[23] Hendry S Pang JB Byrne DJ Lakhani SR Cummings MC Campbell IG. Relationship of the breast ductal carcinoma *in situ* immune microenvironment with clinicopathological and genetic features. Clin Cancer Res. (2017) 23:5210–17. 10.1158/1078-0432.CCR-17-0743 28611201

[24] Beguinot M Dauplat MM Kwiatkowski F Lebouedec G Tixier L Pomel C. Analysis of tumour-infiltrating lymphocytes reveals two new biologically different subgroups of breast ductal carcinoma *in situ*. BMC Cancer. (2018) 18:129. 10.1186/s12885-018-4013-6 29394917 PMC5797400

[25] Qinfeng S Depu W Xiaofeng Y Shah W Hongwei C Yili W. *In situ* observation of the effects of local irradiation on cytotoxic and regulatory T lymphocytes in cervical cancer tissue. Radiat Res. (2013) 179:584–9. 10.1667/RR3155.1 23510275

[26] Liang Y Lü W Zhang X Lü B. Tumor-infiltrating CD8+ and FOXP3+ lymphocytes before and after neoadjuvant chemotherapy in cervical cancer. Diagn Pathol. (2018) 13:93. 10.1186/s13000-018-0770-4 30474571 PMC6260654

[27] Teng F Mu D Meng X Kong L Zhu H Liu S. Tumor infiltrating lymphocytes (TILs) before and after neoadjuvant chemoradiotherapy and its clinical utility for rectal cancer. Am J Cancer Res. (2015) 5:2064–74. 26269765 PMC4529625

[28] Mirjolet C Charon-Barra C Ladoire S Arbez-Gindre F Bertaut A Ghiringhelli F. Tumor lymphocyte immune response to preoperative radiotherapy in locally advanced rectal cancer: The LYMPHOREC study. Oncoimmunology. (2017) 7:e1396402. 10.1080/2162402X.2017.1396402 29399395 PMC5790354

[29] Jarosch A Sommer U Bogner A Reißfelderb C Weitza J Krause M Neoadjuvant radiochemotherapy decreases the total amount of tumor infiltrating lymphocytes, but increases the number of CD8C/Granzyme BC (GrzB) cytotoxic T-cells in rectal cancer. Oncoimmunology. (2017) 7:e1393133 10.1080/2162402X.2017.1393133 29308324 PMC5749657

[30] Denkert C von Minckwitz G Darb-Esfahani S Lederer B Heppner BI Weber KE. n-infiltrating lymphocytes and prognosis in different subtypes of breast cancer: a pooled analysis of 3771 patients treated with neoadjuvant therapy. Lancet Oncol. (2018) 19:40–50. 10.1016/S1470-2045(17)30904-X 29233559

